# Applying a Structural‐Competency Framework to the Implementation of Strategies to Reduce Disparities for Sexual and Gender Minority Youth

**DOI:** 10.1111/josh.12964

**Published:** 2020-11-12

**Authors:** Daniel G. Shattuck, Cathleen E. Willging, Amy E. Green

**Affiliations:** ^1^ Pacific Institute for Research and Evaluation, 851 University Blvd SE, Suite 101 Albuquerque NM 87106; ^2^ The Trevor Project, P.O. Box 69232 West Hollywood CA 90069

**Keywords:** implementation science, health disparities, LGBTQ youth, sexual and gender minority, structural competency, WSCC model

## Abstract

**BACKGROUND:**

Sexual and gender minority youth (SGMY) are at high risk for adverse health outcomes. Safer schools decrease this risk. The US Centers for Disease Control and Prevention has identified 6 practices that can make schools safer for SGMY, yet few US schools implement them all. We apply a structural competency framework to elucidate factors contributing to this implementation gap.

**METHODS:**

We conducted 75 interviews and 32 focus groups with school professionals in 18 New Mexico high schools to assess factors impacting implementation of the practices over 2 years. We analyzed data using iterative coding, thematic identification techniques, and the sensitizing concept of structural competency.

**RESULTS:**

Themes included: rendering an invisible population visible; critical thinking about LGBTQ inequalities; building school personnel capacity; intersecting cultural, religious, and political conflicts; and tackling community‐based sources of stigma and discrimination.

**Conclusions:**

Underlying cultural and structural forces render SGMY invisible and constrain what schools can accomplish. Professional development encouraging critical thinking about structural inequities is foundational, but efforts to close the implementation gap must attend to structural forces producing disparities for SGMY. Structural competency can strengthen the ability of the Whole School, Whole Community, and Whole Child model's cross‐sector coordination of policy and process to meet the needs of every student.

Sexual and gender minority youth (SGMY)—lesbian, gay, bisexual, transgender, or queer (LGBTQ) adolescents—are at elevated risk for adverse health outcomes compared to their straight, cisgender peers, including suicidality, substance misuse, and risky sexual behaviors.^1^, ^2^ These disparities stem from experiences of discrimination and victimization shaped by social structures.^3^ Interventions to reduce these disparities must occur at the individual level (eg, counseling) and the structural level (eg, antiharassment policies). Making schools safer for SGMY can address structural influences on their well‐being and reduce negative outcomes.^1^ The US Centers for Disease Control and Prevention (CDC) collects data on the implementation of 6 school‐based practices considered supportive of SGMY. These practices include identification of safe spaces, ie, designated offices, classrooms, or student organizations, where SGMY can receive support from school staff; prohibition of harassment based on sexual orientation and gender identity; provision of professional development (PD) to school staff on safe and supportive school environments; facilitation of access to providers with experience delivering health services to SGMY; facilitation of access to providers with experience delivering social and psychological services to SGMY; and use of health curricula that includes information relevant to SGMY.^4^

These practices can improve school environments for SGMY and their peers, and positively impact health outcomes^1^, ^5^, ^6^; however, in 2018 only 15.3% of US schools implemented all 6.^4^, ^7^, ^8^ In 2015, we initiated a 5‐year cluster randomized trial called “Implementing School Strategies to Reduce LGBTQ+ Adolescent Suicide,” or RLAS, to address this research‐to‐practice gap.^9^, ^10^ Researchers used a mixed‐method research design to examine use of the supportive practices in New Mexico high schools. Participating high schools were located throughout the state, including in both urban and rural areas. Schools were randomized into “implementation‐support” and “delayed implementation‐support” conditions. Each individual school comprised a cluster in the randomization. Changes in health outcomes at the student population level within each school were assessed using data from the New Mexico Youth Risk and Resiliency Survey, the state‐specific extension of the US Centers for Disease Control and Prevention Youth Risk Behavior Surveillance System.

The 18 implementation‐support schools engaged in the Dynamic Adaptation Process, an iterative and data‐informed implementation science framework, to support Implementation Resource Teams (IRTs) of 4 to 6 persons charged with promoting the uptake of 2 practices annually over 3 years with guidance from a coach.^11^ Those schools randomized into the delayed implementation‐support condition were asked to continue with services as usual. These schools would be provided with implementation support and resources in the final year of the study. More on the protocol and methodologies of the RLAS study has been previously published.^9^

For this qualitative analysis, we applied a structural‐competency framework to elucidate factors affecting practice uptake during the first 2 years of implementation. This framework recognizes that health outcomes are shaped by upstream factors, including systematic social, political, and economic marginalization, stigma, discrimination, a history of diagnosing an LGBTQ people as ill, and violence.^12^, ^13^ Structural competency builds on cultural competency frameworks. Cultural competency focuses on interpersonal strategies to mitigate culturally specific forms of stigma. Structural competency adds to that focus by addressing structural vulnerability and offering methods to decrease disparities at a systemic level.^13^, ^14^, ^15^ This framework advances interventions focused on policies, procedures, and provision of resources for ameliorating adverse health outcomes among vulnerable populations, including LGBTQ people.^13^, ^16^, ^17^ Much structural‐competency research centers on clinical settings, and a focus on criminal justice and drug policy is burgeoning.^13^, ^17^, ^18^, ^19^, ^20^, ^21^ Schools are key components of health and health care systems for youth, as recognized by models like the Whole School, Whole Community, Whole Child (WSCC) approach developed and advocated by many organizations, such as the CDC and the ASCD.^22^ Leveraging structural competency within school health initiatives can effectively address the vulnerabilities of SGMY and impact both health and educational outcomes. Furthermore, whereas there is growing guidance on how to implement the WSCC in regards to entire student bodies,^23^, ^24^ more attention is needed on how these models can benefit specific marginalized and vulnerable populations, like SGMY, for which evidence‐based practices are either limited or limited in their use. For these reasons, research on structurally competent innovations in schools is needed to illuminate how practices can be implemented successfully to reduce disparities.

## METHODS

### Participants

For this qualitative analysis, we recruited a purposive sample of participants from 18 implementation‐support schools during the first and second years of implementation. Researchers invited leads and administrators from each implementation‐support school to participate in individual interviews and the remaining IRT members to take part in focus groups. Leads were self‐identified advocates for LGBTQ+ youth charged with convening IRTs and promoting the 6 practices in schools. Leads included school nurses, social workers, librarians, teachers, and administrative staff. Administrators supported leads and IRTs; these individuals were generally principals and assistant principals. The IRTs members included administrators, school nurses, counselors, social workers, teachers, security staff, and coaches. Further details about the recruitment and purposive sampling methods can be found elsewhere.^25^ A minority of IRTs included students and community members. In both years, participants were mostly women (70–77%) and white (70–73%), with many identifying as Hispanic (37–39%). The majority worked in local schools for several years. Table 1 displays demographic data.

**Table 1:** Participant Demographics

		2018	2019
		N: 81	N: 106
Average Age		45	47
Gender Identity*	Male	15 (18.52%)	30 (28.3%)
	Female	62 (76.54%)	74 (69.81%)
	Trans man	1 (1.23%)	1 (0.94%)
	Trans woman	1 (1.23%)	0
	Gender queer/gender nonconforming	1 (1.23%)	3 (2.83%)
	Other	0	1 (0.94%)
	Prefer not to say	1 (1.23%)	0
Sexual Orientation*	Bisexual	4 (4.94%)	9 (8.49%)
	Heterosexual	64 (9.01%)	81 (76.42%)
	Gay/Lesbian	8 (9.88%)	12 (11.32%)
	Queer	2 (2.47%)	3 (2.83%)
	Questioning	1 (1.23%)	1 (0.94%)
	Other	0	2 (1.89%)
	Prefer not to say	4 (4.94%)	3 (2.83%)
Race*	American Indian	10 (12.35%)	9 (8.49%)
	African American	6 (7.41%)	3 (2.83%)
	Middle Eastern	1 (1.23%)	0
	Native Hawaiian	0	0
	Asian	1 (1.23%)	1 (0.94%)
	White	56 (69.14%)	77 (72.64%)
	Other	14 (17.28%)	18 (16.98%)
Hispanic		30 (37.04%)	41 (38.68%)

* Participants were able to select all options that applied.

### Procedure

We conducted 75 semi‐structured interviews and 32 focus groups during 2018 and 2019. Interviews were about 1 h in length and focus groups about 90 minutes. Open‐ended questions centered on experiences of implementation; factors within school, district, state, and national contexts affecting implementation; practice uptake; and perceived practice impacts.

### Data Analysis

We digitally recorded and transcribed interviews and focus groups. The first author conducted iterative coding using methods of grounded theory.^26^ “Open coding” was used to determine key themes and issues articulated across participant groups. “Focused coding” was then used to refine patterns and determine representativeness. Codes were based on interview topics (eg, “policies relevant to SGMY” and “barriers to addressing SGMY needs”). New codes were identified to capture emergent issues (eg, “LGBTQ visibility” and “physical environment”). Resulting codes and representative text were grouped into broad themes and shared with all authors for review. Finally, we considered the resulting themes in relation to the sensitizing concept of structural competency.^27^ A sensitizing concept is an interpretive device often used within inductive coding processes that guides researchers' attention to important features of or patterns within data.^27^

## RESULTS

### Rendering An Invisible Population Visible

Staff education around LGBTQ issues cast attention upon an otherwise “invisible” population. Participants had minimal awareness of SGMY within their schools prior to RLAS. One teacher explained: “The thing is you think, ‘Oh, that's only 1% of the population.’ Well, we're talking about something that's more 5% to 10% of the population!” This realization came after initial PD oriented school staff to SGMY issues. Most participants described their previous deficit in formal education related to SGMY as a barrier to aiding these students.

Greater cognizance among staff prompted efforts to develop or strengthen school‐based support for SGMY and “normalize” their presence on campus. One teacher described SGMY commending staff for participating in trainings. Some participants also spoke about visibility among LGBTQ adults, contending that having “out” faculty positively influenced campus environments. A teacher explicated: “[A coworker] came out as transgender. That created an awareness. I…[love]…the way she has spoken to the kids.” The normalization of LGBTQ identities reportedly promoted a sense of inclusiveness and safety for SGMY on campus.

### Encouraging Critical Thinking About LGBTQ Inequalities

Participants cited PD as “eye opening” for encouraging critical thinking about SGMY inequities. They reported greater reflection on difficulties confronting SGMY and the dearth of supports to protect them, highlighting discriminatory practices engendering structural vulnerabilities institutionalized in educational settings. Participants pointed to a desire among colleagues to treat all students equally as justification for not providing supports specific to SGMY. For example, many reported beliefs that bullying policies applied to all students and enumeration of groups was unnecessary. However, inadequate attention to SGMY bullying experiences, combined with insufficient school staff preparation to intervene, likely contributed to SGMY feeling more unsafe in schools compared to straight, cisgender peers. Regarding attempts to educate staff, one principal stated the importance of, “getting [faculty] to understand we're not trying to change beliefs…but understanding…how do you work with a student that's being bullied, that feels this way, that has a chance of committing suicide at a higher rate?” This principal worried that personal beliefs could pose obstacles to changing practices on campus contributing to health disparities of SGMY.

Participants credited PD and increased reflection on LBGTQ+ issues for an observed uptick in staff speaking with students or inviting students to meet with IRTs. In one school, the Genders and Sexualities Alliance (GSA) faculty sponsor organized meetings between GSA students and teachers to integrate LGBTQ considerations into health curricula. She explained: “The kids started voicing their concerns, especially around health education, and how they felt it was stigmatized. How they felt like [LGBTQ identity] was treated as Other instead of just a part of the normal.” In addition to undermining assumptions about the inclusivity of curricula, these talks led staff to question their perceptions of student experiences. One principal who previously believed all students felt safe at the school, broke down crying recounting conversations with a transgender student who had experienced violent harassment but had not felt safe reporting the incidences. Professional development coupled with student‐centered dialogs brought invisible SGMY experiences to light, challenging assumptions that treating all students “equally” was best practice.

### Building School Personnel Capacity

Professional development improved school personnel's comfort in talking to and about SGMY, and enhanced school capacity to address SGMY needs. Having colleagues engage in SGMY‐related PD, ask questions, and volunteer for activities signified this increased comfort. One nurse positively described discussions among staff after screening an educational video, appreciating that coworkers felt empowered to share personal experiences and talk openly about sexuality and gender—taboo topics in many school communities. For many, exposure to education and practice with supportive behaviors they could enact individually (eg, sharing pronouns) enhanced comfort.

Building on foundational PD, staff who were most relaxed in speaking with colleagues about LGBTQ issues spearheaded follow‐up education. Consequently, IRTs nurtured cadres of staff who voluntarily positioned themselves as resources. Most schools provided Question, Persuade, and Refer (QPR) Suicide Intervention trainings to staff, supplementing existing suicide protocols by dispersing responsibility for intervention among all school personnel. A similar internal network building was achieved through Safe Zones, whereby volunteers could enlist each other for advice in assisting SGMY. Safe Zones is an intentional training program designed to recognize volunteers as “safe” people or resources for marginalized students, including SGMY; staff can communicate their participation to students and other staff through the use of visual markers such as rainbow flags or posters in their classrooms, offices, or other spaces.^28^

School personnel reported increased awareness of resources as a boon to their comfort in engaging SGMY. They learned about unfamiliar resources and how to approach familiar resources with a new focus. A nurse expressed surprise at learning about services at a well‐known resource: “I was amazed to discover our school‐based health clinics will do hormone treatment.” Until her involvement in IRT, she had not considered where students could get LGBTQ‐affirmative health care.

Partnering with community resources was key to participants attempting to overcome structural barriers thwarting SGMY access to health and psychological services. The limited service capacity of schools was complemented by an increased ability among school personnel to refer students to community resources for unmet needs. However, resource availability was dependent on broader community environments. About 44% of implementation‐support schools had on‐campus school‐based health centers (SBHCs) providing some LGBTQ‐competent clinical care, including reproductive health and psychological services. Schools without SBHCs relied on community resources, including public health offices which provided access to sexual health prevention, testing, and intervention services. Personnel in rural schools found it difficult to refer students to community providers because they did not exist or were not easily accessible due to transportation barriers. School personnel were also often unsure about the LGBTQ inclusivity of available resources. In these cases, IRTs used statewide databases of providers with LGBTQ‐inclusive clinical practices hosted by local LGBTQ advocacy organizations.

Some IRTs tried improving access by allowing students to obtain information directly themselves, rather than through school health professionals, with the goal of increasing recommended resource usage. In multiple schools, IRTs distributed printed materials with crisis hotlines, including inside restroom stalls for private consultation, and in one case, adding QR or other graphic codes to posters that students can scan with smartphone cameras to access online resources.

### Incorporating Structural Innovations

School personnel championed structural innovations to cultivate inclusive environments. Innovations addressing LGBTQ‐related stigma and SGMY needs included changes to physical spaces, policies, student activities, and curricula, and visible markers of LGBTQ inclusivity. For instance, to increase access and safety of restrooms for transgender students, IRTs led efforts to transition from gendered to single‐user restrooms. A social worker described how such a structural innovation benefited a range of students: “We put in a bathroom lock, so we have it for any student, LGBTQ, student with a disability, whatever. You punch in a number, and you can go in.” The lock selected for this restroom allowed for discreet access and security, decreasing the likelihood of students being “outed” for requesting keys to use it.

Such accommodations were integrated into a wider innovation adopted by some schools: individual support or gender support plans. These plans outlined accommodations, such as using chosen names, for transgender students in consultation with youth, guardians, and school representatives. Only one district had such plans available prior to RLAS, while administrators and staff in most others knew nothing about such an innovation. One teacher explained, “Until 2 weeks ago, we didn't know that was even a thing. It sets the tone. These are the pronouns of request.” As she noted, these binding agreements set the groundwork for recognizing students' gender identities and providing equitable treatment. In several schools where IRTs established protocols for such plans, staff reported their almost immediate adoption.

The IRTs also focused on establishing and expanding GSAs, with 15 implementation‐support schools having GSAs at the end of the second year of implementation. For some schools with pre‐existing GSAs, IRT members followed student direction and supported the expansion of their reach and missions. One GSA sponsor explained students' desire to transition from socializing to focusing on outreach: “They want us to expand our advocacy work, beyond just LGBTQ to others.” To support such missions, some clubs brought in new staff sponsors whose interests aligned with theirs, or who appealed to underrepresented groups. A second GSA sponsor described engaging a Spanish‐speaking staff member in the club to engage persons of Mexican heritage. Overall, the visibility of these student organizations increased, with GSAs participating in school‐wide events, for example, by entering floats in homecoming parades. Several GSAs also participated in GLSEN's Day of Silence, a national student‐led demonstration where students and allies take a vow of silence to protest harassment and discrimination of LGBTQ people in schools.^29^

Some IRTs focused on eliminating seemingly innocuous institutionalized activities that alienated SGMY. One IRT sought to remove “Powder Puff Football” and “Gender Swap Day” from their Homecoming Spirit Week. While many staff at first believed these activities were harmless, their discussions led them to realize the exclusionary politics undergirding them. In these instances, it was appropriate for straight, cisgender students to behave and dress in a manner poking fun at gender, while SGMY identities were policed for adherence to those same norms at risk of punishment or, worse, violence.

In addition to consulting SGMY on the deficits in curricula, some IRTs addressed increasing inclusivity and comprehensiveness of health curricula by pursuing avenues for PD specific to health education. Other IRTs invited local experts, like Planned Parenthood health educators, to present sexual and reproductive health education. In schools offering online health education, IRTs vetted the appropriateness of courses.

Several IRTs focused on school libraries as important SGMY resources. Participants perceived libraries as a refuge, citing the tendency of students who “didn't fit in” to eat lunch there. In libraries, students could access educational texts that supplemented official health curricula, receive support from a librarian who was also a Safe Zone volunteer, and see LGBTQ identities reflected decorations or books. These innovations allowed students to obtain information and examples of LGBTQ representation otherwise unavailable.

### Intersecting Cultural, Religious, and Political Conflicts

Participants described conflicts within communities that undermine SGMY support. Communities were characterized as “very traditional” or influenced by “machismo.” One social worker described local gender norms as outdated: “The biggest barrier is the old mindset Girls should be with boys; boys should be with girls.” Lamenting family dynamics spurred on by traditional views, one teacher said, “We cannot control what is being taught at home. That culture of machismo is preventing people from just loving everybody. You can't control that.” Participants discussed how these views might negatively affect LGBTQ people.

The influence of gender norms in communities shaped school environments, particularly regarding sports. One nurse explained: “It's even coming from the coaches. It's like saying, ‘Oh, you are throwing like a girl.’ It's like, well, ‘Don't say that.’ Why don't you just say, ‘You threw really bad?’ Just say what you mean.” She went on to explain that these attitudes would be hard to change because of roots in the community's culture.

Religion influenced acceptance of LGBTQ people locally. A social worker stated that community members invoked religion to justify opinions about gender and sexuality saying, “It's always ‘That's a sin, it's wrong.’” The perceptions of religious peers and relatives also perturbed participants. During a focus group 2 IRT members expressed dismay after their “very religious” relatives disapproved of efforts to make their school safer for SGMY.

The political climates of communities posed obstacles, with participants citing political beliefs among their coworkers and negative interactions among student peers as impediments to change. One nurse commented: “With the current political environment, I've seen negative discourse. Students almost feel more permission to be nasty to each other.” Another nurse described students loudly expressing anti‐immigrant sentiments. Such discourses contributed to hostile environments in which many students felt unsafe, including SGMY.

Participants acknowledged that navigating communities intolerant or unsupportive of diverse sexualities or genders could stress school personnel involved in implementing the 6 school practices. Personal conflicts prevented some from committing to implementation. One nurse described conflicts hindering staff from recognizing publicly their support of SGMY through participation on an IRT: “[I told her] ‘You're already doing it. It's already a safe haven.’ She said, ‘I don't want it conflicting with my religion.’ She's just afraid to commit because of a church.” Community‐rooted stigma and conflict rarely remained outside school walls.

### Tackling Community‐Based Sources of Stigma and Discrimination

Participants characterized communities as sources of stigma and discrimination of SGMY at schools. First, lack of family support was considered a barrier that could not be ignored. A school nurse talked of problems extending from families and the school's ability to intervene:

*One barrier is parents disowning kids, throwing them out of the house…. They need to know that this is who your child is. I'm hoping we can open up the door to parents to say, “Feel safe to ask questions. Don't think you're going to be pointed out, because your child is not the child you thought they were… This is your child. You have to love them*.”

Similarly, a teacher described her efforts to support a student in managing her family's reaction to her coming out as lesbian: “Her parents ended up finding out. [It's not] resolved yet but it's something I'm working with the student to help manage.”

Community input on school policies was another issue requiring navigation. One principal observed: “A lot of people opposed even having gender‐neutral bathrooms at schools. It seems like people are pushing their own agendas and…it affects the policies being put on the table and the policies that go into effect.” Community input, as cited by this principal, was channeled through multiple levels including the school board, district‐level leadership, and direct engagement of parents with school administration.

Despite these sources of struggle, participants suggested their schools were a place to start dismantling barriers. The school was a catalyst for wider change, as a principal explained:

*I can make it…acceptable on this campus to not only have the conversation, but to embrace the kid so when those conversations are happening at home, I've got students who say, “What are you talking about? I know this kid. He's awesome.” Those dinner conversations are powerful in families. We can only control so much, but if I can build a climate here of acceptance, that will bleed out into the community*.

Participants hoped their schools could serve as safe places for SGMY to find acceptance and support lacking in their homes or communities, and that these institutions could galvanize wider change by shifting attitudes of students and empowering them to stand up for each other.

## DISCUSSION

Professional development was a first step toward implementing the 6 practices and cultivating structural competency in schools. Education including information on terminology or language associated with LGBTQ populations was beneficial for staff and built critical awareness of SGMY presence in schools, combating the common misperception that these youth did not exist.^25^ Trainings were reportedly impactful when they described SGMY disparities and prompted new thinking about underlying social, political, and economic influences. This deeper understanding shifted attention from effecting individualized behavioral changes to advocating for and enacting institutional changes.

Structural competency proponents assert that addressing the political, economic, and social systems perpetuating disparities requires interventions at institutional levels.^12^, ^13^, ^14^, ^15^, ^17^ School staff implemented several practices at such levels by changing policy and health curricula, establishing internal support systems (eg, Safe Zones), and modifying physical environments (eg, designating single‐user restrooms). Staff capacity‐building complemented institutional‐level efforts. Skill‐building education, such as QPR trainings, augmented abilities to recognize signs of suicidality, intervene, and facilitate access to supports. Notably, how schools aided students in accessing health care exemplified the integration of individual capacity‐building and institutional changes. Staff developed knowledge about providers by attending to their SGM‐friendliness and made this information accessible to students and coworkers. Referral lists, school websites, and print materials ensured student and staff access to this information, thereby enhancing schools' ability to function as an access point.

Much of what schools accomplished was managed through careful connection to intermediary resources. From PD strengthening staff capacity to accessing community‐sourced data concerning LGBTQ‐friendly services, participants partnered with state agencies and LGBTQ advocacy organizations. In clinical environments, forging connections to external resources and having staff knowledgeable about resources facilitates social and economic support alongside treatment for medical issues.^16^ In schools, a similar combination of institutional‐level connection and individual knowledge can produce the same capacity, improving their overall ability to serve SGMY as a part of health care systems.

The importance of schools as a support for SGMY and their facilitation of access to external resources is underscored by the recurrent theme of schools as safe spaces in unsupportive wider communities. Participants believed schools needed to overcome the absence of family support. In terms of suicidality, school connectedness is second to family connectedness as a protective factor.^30^ Considering that schools are situated in contexts where community health care resources are limited or their LGBTQ competency is questionable, schools become central nodes in health care systems for support and access otherwise non‐existent for some youth.

### Limitations

This study is limited to high schools in a single state in the Southwest United States, which may limit its generalizability. The purposeful sampling strategy may lead to an overrepresentation of school personnel concerned about the well‐being of SGMY, or with vested interests in portraying themselves and their schools positively. Further research must address intersectional issues related to other axes of difference (eg, race and class) affecting practice implementation.

## CONCLUSIONS

This study points to structural competency's role in addressing SGMY disparities in schools. Encouraging cultural competency and individual interventions is important, but alone is unlikely to result in enduring change. Education encouraging critical thinking about structural inequities is foundational to enhancing staff capacity. Institutional‐level changes can enable ongoing support within schools so that structural vulnerabilities reinforcing disparities are recognized and addressed, while minimizing harms attributable to individual intolerance and staff turnover.

Second, when implementing new programs, attention to structural competence throughout implementation processes can reduce chances that innovations perpetuate inequities. Educational opportunities for staff promoted SGMY visibility and legibility of their needs, undermining perspectives that all students were or should be treated the same. The goal of structural competency is to work toward equity by providing supports that address the different needs of disadvantaged groups. Ignoring existing inequities while attempting to implement new policies or practices runs the risk of those innovations perpetuating or exacerbating disparities.

Third, schools are critical sites for applying structural competency as they are sites of early identification and prevention and a de facto part of health care systems. If LGBTQ populations are afforded supports in these institutions that address structural vulnerabilities and reduce stigma, both health and educational outcomes can be improved.^1^, ^5^, ^6^

Finally, the structure of institutions (eg, schools) must be changed from within and through purposeful engagement with community‐based resources. Staff described schools as bastions against the adversities of the outside world, as schools provided a much‐needed safe space for SGMY. It is up to people in these institutions to lead changes for the betterment of their environments and the safety of students, regardless of oppositional views from the outside. Yet, without partnering with community organizations and resources, many changes, including the ability to refer to LGBTQ appropriate services, would not have been implemented in the schools.

### IMPLICATIONS FOR SCHOOL HEALTH

This study can inform use of WSCC. First, while this model encourages coordination of policies and procedures across sectors, it does not offer guidance on how to implement such coordination.^31^ Implementation science studies, such as RLAS, illuminate strategies schools use when implementing health programs. Attention to implementation frameworks can assist staff in tailoring abstract guidance to schools in a structured and feedback‐informed way, improving the fit of practices to these contexts and the efficiency with which WSCC guidance can be put into place. Second, a structural competency framework and models such as the WSCC can work together to comprehensively improve school cultures and climates at a foundational level. A structural competency focus builds on the cross‐sector coordination of policy and process advocated under WSCC, attuning the innovations schools make to the structural vulnerabilities of SGMY. As the WSCC emphasizes and the RLAS study demonstrates, multiple, if not all, sectors of schools must be involved in institutional innovations to address the needs of students in order to enact wide‐spread and lasting change. To merge a structural competency framework with the WSCC, we must also attend to the potential divergence of schools and communities concerning supports for marginalized populations like SGMY, which could be problematic for a model advocating for school‐community coordination. The LGBTQ‐related stigma undermining community support must not inform the capacity, policies, and procedures of schools. By institutionalizing LGBTQ inclusive policies and procedures, schools can ensure a social and emotional climate that promotes positive health outcomes for SGMY, provides and set examples for communities.

### Human Subjects Approval Statement

This study was approved by the Institutional Review Board at the Pacific Institute for Research and Evaluation (IRBNet ID # 787984–3).

### Conflict of Interest

All authors of this article declare they have no conflicts of interest.
